# Left thigh phlegmon caused by *Nocardia farcinica* identified by 16S rRNA sequencing in a patient with Leprosy: a case report

**DOI:** 10.1186/1471-2334-13-162

**Published:** 2013-04-04

**Authors:** Pasquale De Nardo, Maria Letizia Giancola, Salvatore Noto, Elisa Gentilotti, Piero Ghirga, Chiara Tommasi, Rita Bellagamba, Maria Grazia Paglia, Emanuele Nicastri, Andrea Antinori, Angela Corpolongo

**Affiliations:** 1 “Lazzaro Spallanzani” National Institute for Infectious Diseases-IRCCS, Via Portuense 292, Rome, 00149, Italy; 2 Azienda Ospedaliera Universitaria “San Martino”, Largo R. Benzi 10, Genoa, 16132, Italy

**Keywords:** *Nocardia*, Hansen’s disease, Phlegmon

## Abstract

**Background:**

In recent years, *Nocardia farcinica* has been reported to be an increasingly frequent cause of localized and disseminated infections in the immunocompromised patient. However, recent literature is limited. We report a case of left thigh phlegmon caused by N. *farcinica* that occurred in a patient with Leprosy undergoing treatment with prednisone for leprosy reaction.

**Case presentation:**

We describe the case of left thigh phlegmon caused by *Nocardia farcinica* in a 54-year-old Italian man affected by multi-bacillary leprosy. The patient had worked in South America for 11 years. Seven months after his return to Italy, he was diagnosed with Leprosy and started multi-drug antibiotic therapy plus thalidomide and steroids. Then, during therapy with rifampicin monthly, minocycline 100 mg daily, moxifloxacin 400 mg daily, and prednisone (the latter to treat type 2 leprosy reaction), the patient complained of high fever associated with erythema, swelling, and pain in the left thigh. Therefore, he was admitted to our hospital with the clinical suspicion of cellulitis. Ultrasound examination and Magnetic Resonance Imaging showed left thigh phlegmon. He was treated with drainage and antibiotic therapy (meropenem and vancomycin replaced by daptomycin). The responsible organism, *Nocardia farcinica*, was identified by 16S rRNA sequencing in the purulent fluid taken out by aspiration. The patient continued treatment with intravenous trimethoprim/sulfamethoxazole and imipenem followed by oral trimethoprim/sulfamethoxazole and moxifloxacin. A whole-body computed tomography did not reveal dissemination to other organs like the lung or brain.

The patient was discharged after complete remission. Oral therapy with trimethoprim/sulfamethoxazole, moxifloxacin, rifampicin monthly, clofazimine and thalidomide was prescribed to be taken at home. One month after discharge from the hospital the patient is in good clinical condition with complete resolution of the phlegmon.

**Conclusion:**

N. *farcinica* is a rare infectious agent that mainly affects immunocompromised patients. Presentation of phlegmon only without disseminated infection is unusual, even in these kinds of patients. In any case, a higher index of suspicion is needed, as diagnosis can easily be missed due to the absence of characteristic symptoms and the several difficulties usually encountered in identifying the pathogen.

## Background

Nocardiosis is a localized or disseminated uncommon infection caused by an aerobic *Actinomycetes* of the genus *Nocardia*[1]. Approximately 50 species of *Nocardia* have been characterized [2], with at least 16 species being recognized as human pathogens [3]. These organisms are opportunistic pathogens affecting immunocompromised hosts, including those with long-term corticosteroid exposure, malignancy, human immunodeficiency virus infection, and a history of transplantation [4]. Earlier reports estimated the occurrence of 500–1,000 new cases each year in the USA, and 150–200 and 90–130 in France and Italy, respectively [5-7]. *N. asteroides*, *N. farcinica* and *N. nova* caused approximately 85% of the total cases [8-10]. An increase in the diagnosis of *N. farcinica* infection is due to the growing population of immunocompromised hosts and improved methods for detecting and identifying the *Nocardia* species. This microorganism can cause localized or disseminated infections that can be life-threatening without prompt diagnosis and proper treatment; furthermore, it is characteristically resistant to multiple anti-microbial agents, including third-generation cephalosporin [1,11].

We report the case of left thigh phlegmon caused by *N. farcinica* that occurred in a patient affected by Leprosy undergoing treatment with prednisone for leprosy reaction.

## Case presentation

A 54 year old Italian man was admitted to the “Lazzaro Spallanzani” National Institute for Infectious Diseases of Rome because of fever, cutaneous lesions and sensory loss of peripheral nerves. He had worked in Venezuela for eleven years. A histological examination of a skin biopsy showed the presence of *Mycobacterium leprae* and the patient was diagnosed as borderline lepromatous (BL) leprosy complicated by erythema nodosum leprosum (ENL) reaction. His bacteriological index (total bacterial load) was 4.17+ (normal range 0-6+) and the morphological index (percentage of bacilli uniformly stained) 0.5%. He was treated with rifampicin monthly and minocycline and moxifloxacin daily. The ENL was recurrent and treated with oral prednisone (75 mg) and thalidomide. Sixteen months after starting therapy for Leprosy (and after about a year of steroid therapy), the patient started to complain of high fever (39°C) associated with erythema, swelling and pain of left thigh, and he was admitted to our hospital for the second time with suspicion of cellulitis. He referred that the symptoms had started about 7 days before admission, but he denied a history of local trauma or recreational drug abuse. Diabetes mellitus had been diagnosed two months before.

**Figure 1:**
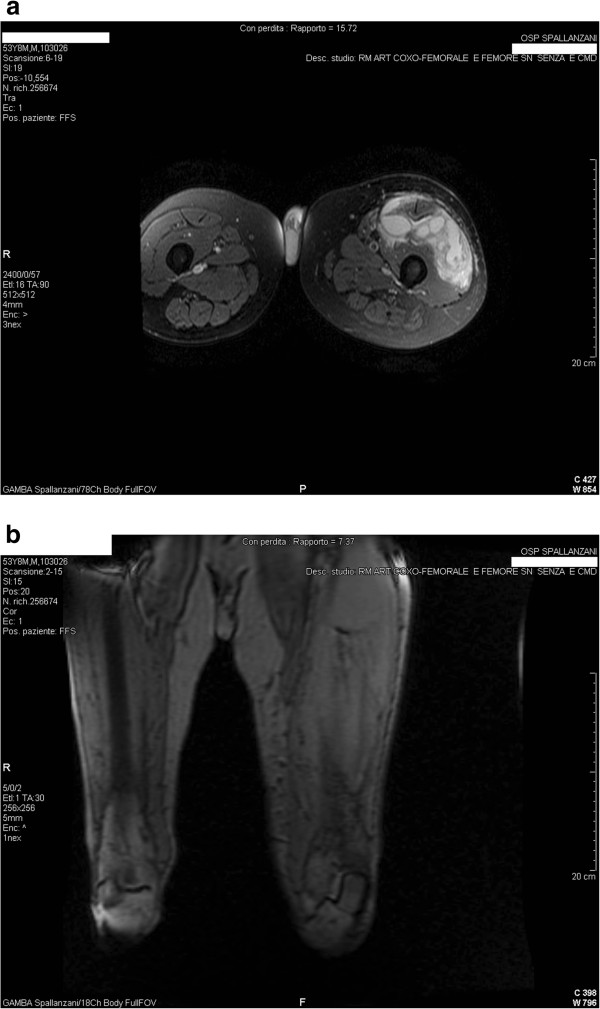
**Magnetic Resonance Imaging of left thigh at admission. a**. T1 after contrast coronal image showing hyperintense necrotic lesion of the left thight; **b**. T2 FLAIR weighted sagittal image of the same lesion that appear hypointense.

On physical examination, his vital signs were as follows: temperature 39°C, blood pressure 130/80 mmHg, pulse rate 95 beats per minute, and respiratory rate 15 breaths per minute. Laboratory examinations revealed: white blood cells (WBC) 14,500/μL (4,3-10,8) (87% neutrophils, 4.1% lymphocytes), haemoglobin 11 g/dL (12–18), platelets 234,000/μL (80,000 – 400,000), C-reactive protein (CRP) 32 mg/dL (0–0.16), and erythrocyte sedimentation rate (ESR) 82 mm/h (0–15). Chest X-ray was normal. Ultrasound examination and Magnetic Resonance Imaging (MRI) of the lower extremities revealed left thigh phlegmon (Figure 1). Ultrasound guided incision and drainage were performed at the bedside with leaking, copious amounts of pus. Samples were sent to the laboratory for Gram and acid -fast bacilli staining, cultures, and PCR amplification of bacterial DNA. Furthermore, more sets of blood cultures were performed.

Intravenous vancomycin and meropenem were started: 500 mg every six hours, and 1 g every 8 hours, respectively, and moxifloxacin and minocycline were stopped. Incision was repeated on hospital day 3 and vancomycin was replaced by daptomycin for the persistence of high fever; prednisone was reduced to 50 mg. After 96 hours of incubation, blood cultures and aerobic and anaerobic cultures of the sample were negative. On day 6, the patient had type 2 leprosy reaction and prednisone was again increased to 75 mg. Seven days after initiating daptomycin, an ultrasound examination showed the first decrease of both phlegmon volume and peripheral oedema.

**Figure 2:**
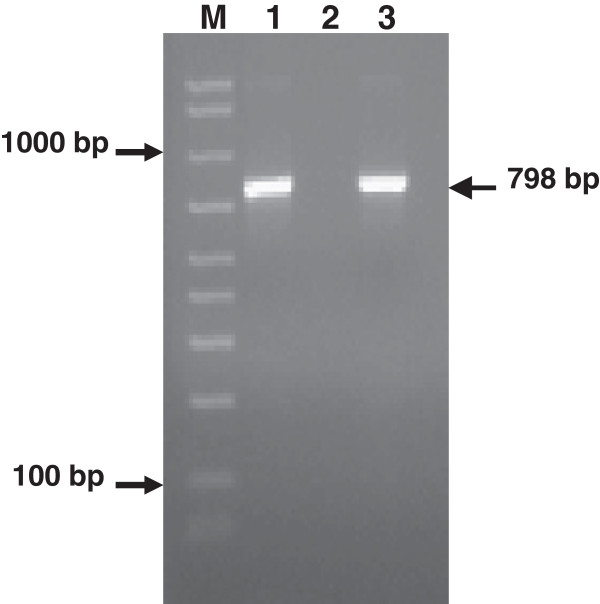
**Two percent agarose gel electrophoresis showing the product of amplification of the 798-bp fragment of the 16S rRNA gene.** DNA sequencing of such a fragment further allowed the identification of *Nocardia farcinica.* Lane 1 = PCR product obtained from a phlegmon sample of our patient; lane 2 = negative control (water); lane 3 = positive control (*Streptococcus pneumoniae* ATCC 49619); M = DNA size reference marker (50-2000-bp Amplisize, Biorad).

On hospital day 15, the pathogen was identified as *N. farcinica* by 16S rRNA gene sequencing in the purulent fluid, whereas the cultures were negative. Amplified PCR products (Figure 2) were purified and directly sequenced. Related DNA sequences were searched in GenBank by using the BLAST (Basic Local Alignment Search Tool) server. The most closely related sequences belonged to *Nocardia farcinica* species. The further identity of the pathogen was also confirmed by sequencing a region of 16S rRNA gene of Nocardia with a PCR that used genus-specific primers [12]. Sequence data of the amplified PCR products showed great identity (99%) to the species *Nocardia farcinica*

**Figure 3:**
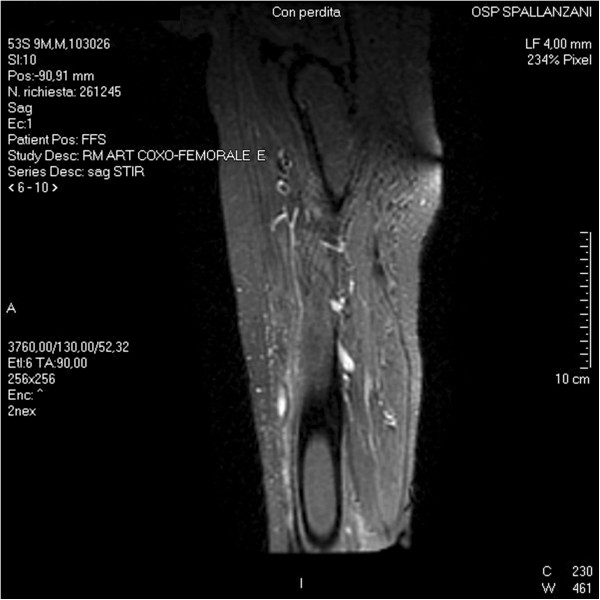
Magnetic Resonance Imaging performed after a month of antibiotic therapy shows complete resolution of the phlegmon.

The patient was shifted to a therapy based on intravenous trimethoprim/sulfamethoxazole and imipenem for 1 month. A whole-body computed tomography (CT-scan) revealed no dissemination to other organs like the lung or brain. On hospital day 22, prednisone was stopped and the leprosy reaction was treated with clofazimine and thalidomide. After a month of therapy, MRI revealed a decreased amount of phlegmon without osteomyelitis of the left femur (Figure 3).

The patient was discharged after complete remission at day 51 with a prescription of oral trimethoprim/sulfamethoxazole, moxifloxacin, rifampicin monthly, clofazimine and thalidomide.

One month after discharge from the hospital the patient is in good clinical condition.

## Conclusion

*N.farcinica* is a Gram–positive, branching, filamentous bacillus causing many localized and disseminated infectious in humans, including pulmonary and wound infections, brain abscesses, and bacteraemia. Immunocompromised hosts and patients undergoing immunosuppressive therapies are more commonly affected by this bacterium [2].

Anton Y. Peleg *et al.* described the risk factors of *N. farcinica* infection in organ transplant recipients. In this case series, patients with *Nocardia* infection had significantly higher prednisone dosages (p 0.007), lower lymphocyte counts (p 0.003), and higher neutrophil counts (p 0.006) at the time of infection, compared with control subjects [13]. In our case, the patient had undergone therapy with prednisone in the preceding 12 months because of leprosy reaction. At admission he had WBC 14,500/μL with neutrophils 87% (11800/mmc), lymphocytes 4.1% (500/mmc), CD4 cells 34.6% (463/mmc), CD8 cells 24.8% (322/mmc).

The relative frequency of infections caused by the different *Nocardia* species are difficult to determine retrospectively and may vary geographically*. N. farcinica* has been reported to constitute 13,8% and 19% of *Nocardia* isolates in Italy from 1982 to 1992 [14] and from 1993 to 1997 [7], respectively. It is not possible to estimate the actual number of Italian cases due to the potential bias of referrals to public health authorities and also because *Nocardia* strains are not collected at the National Reference Centre.

The clinical manifestations of *N. farcinica* are different. The lung is the most common site of infection (43%). Torres *et al.* observed infections of cutaneous and sub-cutaneous tissues in 10% of cases. In the case of disseminated infections, it was possible to detect a metastatic focus or, more frequently, a primary lesion after traumatic injury. In other cases, the patients had sub-clinical or transient primary pulmonary infection followed by dissemination to the brain, skin, bones, and kidney [15]. In our case, as in the 7/26 cases described in an Italian report about human Nocardiosis between 1993 and 1997 [7], it was not possible to observe previous pulmonary involvement.

Concerning the epidemiology*, Nocardiae* are ubiquitous in the environment and can be found worldwide in fresh and salt water, in a variety of soil types, dust, decaying vegetation, and decaying faecal deposits from animals [2]. Bittar *et al.* described a *Nocardia* infection in a man who was an active gardener [16]. Our patient had gardening as a hobby, which led us to speculate that he could have acquired the infection in this way.

Unfortunately, we did not identify the organism with the cultures and could not perform susceptibility testing. However, previous studies have identified multi-drug-resistance patterns in isolates of *N. farcinica,* characterized by resistance to most beta-lactam antibiotics [17] and susceptibility mainly to amikacin, imipenem, trimethoprim/sulfamethoxazole, and fluoroquinolones [2]. Hansen G. *et al.* reported that fluoroquinolones had variable activity against clinical strains of *Nocardia*, and the newer fluoroquinolones such as moxifloxacin, gatifloxacin, and gemifloxacin, had increased *in vitro* activity compared to ciprofloxacin [17].

Laboratory identification and speciation for *Nocardia* are challenging, and the methodology continues to evolve. Before 2000, *Nocardia* isolates were identified primarily through phenotypic methods. In 2000, the identification of *Nocardia* was enhanced through the use of 16SrRna gene sequencing in research centres [18-20]. In the reported case, it is interesting to underline that our patient presented clinical and radiological improvement after the initiation of therapy with daptomycin. Since no cases of *N. farcinica* treated with daptomycin have been described in literature (there are only sensitivity studies *in vitro*) [21], we started empirical antibiotic combination therapy with imipenem plus trimethoprim-sulfamethoxazole. Information on the duration of treatment is inadequately documented in most reports, varying from 3 to 12 months with a median of 4 months. Primary cutaneous Nocardiosis may be treated with therapy for 2 to 4 months, but only after bone involvement has been excluded [4,6].

In summary, Nocardiosis remains a severe and sometimes potentially life-threatening complication in the immunocompromised host. It is relevant to consider this microorganism in the differential diagnosis of infections, even in cases with unusual presentations such as this one. Because of the aggressiveness, tendency to disseminate, and resistance of the organism to antibiotics, delayed diagnosis can have serious consequences.

### Consent

Written informed consent was obtained from the patient for publication of this case report and any accompanying images. A copy of the written consent is available for review by the Editor-in-Chief of this journal.

## Abbreviations

WBC: White blood cells; CRP: C-reactive protein; ESR: Erythrocyte sedimentation rate; MRI: Magnetic resonance imaging; CT: Computed tomography

## Competing interests

The authors declare that they have no competing interests.

## Authors’ contributions

AC and PDN took care of the patient and wrote the case report; MGP did the laboratory work; MLG and PG took care of the patient; EG, CT, RT took part in the drafting; SN, EN and AA contributed to literature search and reviewed the final manuscript; all authors read and approved the final version of the manuscript.

## Pre-publication history

The pre-publication history for this paper can be accessed here:

http://www.biomedcentral.com/1471-2334/13/162/prepub
